# Supplement Use and Dietary Sources of Folate, Vitamin D, and *n*-3 Fatty Acids during Preconception: The GLIMP2 Study

**DOI:** 10.3390/nu10080962

**Published:** 2018-07-25

**Authors:** Moniek Looman, Claudia van den Berg, Anouk Geelen, Rahul A. K. Samlal, Rik Heijligenberg, Jacqueline M. T. Klein Gunnewiek, Michiel G. J. Balvers, Caroline L. Leendertz-Eggen, Lia D. E. Wijnberger, Edith J. M. Feskens, Elske M. Brouwer-Brolsma

**Affiliations:** 1 Division of Human Nutrition, Wageningen University, P.O. Box 17, 6700 Wageningen, The Netherlands; claudia.vandenberg@wur.nl (C.v.d.B.); anouk.geelen@wur.nl (A.G.); edith.feskens@wur.nl (E.J.M.F.); elske.brouwer-brolsma@wur.nl (E.M.B.-B.); 2 Department of Gynaecology and Obstetrics, Hospital Gelderse Vallei Ede, P.O. Box 9025, 6710 HN Ede, The Netherlands; SamlalR@zgv.nl (R.A.K.S.); LeendertzC@zgv.nl (C.L.L.-E.); 3 Department of Internal Medicine, Hospital Gelderse Vallei Ede, P.O. Box 9025, 6710 HN Ede, The Netherlands; HeijligenbergR@zgv.nl; 4 Clinical Chemistry and Haematology Laboratory, Hospital Gelderse Vallei Ede, P.O. Box 9025, 6710 HN Ede, The Netherlands; KleinGunnewiekJ@zgv.nl (J.M.T.K.G.); BalversM@zgv.nl (M.G.J.B.); 5 Department of Obstetrics and Gynaecology, Rijnstate Hospital, P.O. Box 9555, 6800 Arnhem, The Netherlands; LWijnberger@rijnstate.nl

**Keywords:** preconception, diet, folate, vitamin D, *n*-3 fatty acids, supplements

## Abstract

An adequate nutritional status during the preconception period is important, particularly for folate, vitamin D, and *n*-3 fatty acids (i.e., EPA+DHA). We aimed to determine supplement intake and the main dietary sources of folate, vitamin D, and EPA+DHA using the data of 66 Dutch women aged 18–40 years who wished to become pregnant. Additionally, associations of these intakes with their blood levels were examined. Dietary intake was assessed with a validated food frequency questionnaire, and supplement use with a structured questionnaire. 25-hydroxyvitamin D levels were determined in serum and folate and phospholipid EPA+DHA levels in plasma. Partial Spearman’s correlations, restricted cubic splines and trend analyses over tertiles of nutrient intakes were performed to examine intake-status associations. A large proportion of women did not meet the Dutch recommended intakes of folate (50%), vitamin D (67%), and EPA+DHA (52%). Vegetables were the main contributor to dietary folate intake (25%), oils and fats to dietary vitamin D intake (39%), and fish to dietary EPA+DHA intake (69%). Fourteen percent of the women had an inadequate folate status and 23% an inadequate vitamin D status. Supplemental folate intake, supplemental and dietary vitamin D intake and dietary EPA+DHA intake were significantly associated with their blood levels. In conclusion, even in our highly educated population, a large proportion did not achieve recommended folate, vitamin D and *n*-3 fatty acid intakes. Promotion of folate and vitamin D supplement use and fish consumption is needed to improve intakes and blood levels of these nutrients in women who wish to become pregnant.

## 1. Introduction

A woman’s dietary intake and nutritional status before conception and during pregnancy are important determinants of maternal and foetal health, and child’s health later in life [1,2,3]. Consequently, maternal dietary habits during the preconception period are considered increasingly important to cover the nutritional needs of the foetus and placenta during pregnancy [4], and to optimize maternal and infant health [5]. To promote the health of prospective parents, the Health Council of the Netherlands has published preconception care guidelines [6]. Despite these guidelines, recent studies showed that preconception dietary intake is still suboptimal with high prevalence of inadequate habitual diet and nutrient status [5,7].

Preconception care dietary guidelines including recommendations on optimal intakes of folate, vitamin D, and fish (i.e., *n*-3 fatty acids eicosapentaenoic acid (EPA) and docosahexaenoic acid (DHA)). Recommendations regarding folate intake are predominantly based on the knowledge that an adequate folate intake in the preconception period reduces the risk of a neural tube defect [8]. Although folate naturally occurs in many dietary sources, such as green leafy vegetables, fruits, meat, and dairy products, it is difficult for Dutch women to obtain the recommended amounts of folate through diet alone [6]. Therefore, a daily folic acid supplement (≥400 μg folic acid) is recommended as part of routine preconception and antenatal care [6].

An adequate preconception vitamin D status is important as vitamin D deficiency in the mother has been linked to various issues in the pregnancy such as preeclampsia [9] and gestational diabetes [10] and in their offspring such as low birth weight [11], poor bone growth [12], and an increased risk of recurrent wheeze or asthma [13,14]. Sunlight is the predominant source of vitamin D [15]. Whereas dietary vitamin D can only be obtained through the consumption of a limited number of foods, such as fatty fish, fat spreads, oils, liver, meat, eggs, and dairy products. Therefore, vitamin D supplement use (i.e., 10 µg/day) is recommended for Dutch women who wish to become pregnant and pregnant women, particularly during winter [16].

The *n*-3 fatty acids EPA and DHA are considered to be important nutrients for the foetal brain and retina development [17]. Therefore, women are recommended to consume 200 mg/day EPA+DHA [18], which translates into at least one portion of fatty fish/week [19].

Despite these recommendations, little is known about current intake levels of folate, vitamin D, and EPA+DHA among Dutch women who wish to become pregnant. Moreover, there is limited data on the relative contribution of these nutrients from dietary sources in relation to their blood levels in preconception women. More knowledge on these aspects may benefit further specification of the dietary recommendations in this field of practice. To address this perceived knowledge gap, we aimed to: (i) describe current intake levels of folate, vitamin D, and the *n*-3 fatty acids EPA+DHA from dietary sources and supplements; (ii) determine the relative contribution of the top-5 of dietary sources to the total intake of these nutrients; (iii) examine how dietary and supplementary intakes of these nutrients relate to their blood levels; and (iv) identify which sources contribute the most to the nutritional status per nutrient.

## 2. Materials and Methods

### 2.1. Study Population

This cross-sectional study was performed using baseline data of the Gestational diabetes and Lifestyle of Mothers during Pregnancy 2 (GLIMP2) study; an observational prospective cohort study designed to assess the role of diet, nutrient status, and other risk factors in the development of gestational diabetes mellitus (GDM). Participants were recruited at the Department of Gynaecology and Obstetrics and Department of Internal Medicine at three non-university hospitals in the eastern part of The Netherlands: Gelderse Vallei (Ede); Rijnstate (Arnhem); and Slingeland (Doetinchem). Main inclusion criteria were age between 18 and 40 years, willingness to get pregnant within one year at the time of recruitment, and competent to make their own decisions. Women with a higher risk of developing GDM (i.e., previous pregnancy with GDM or macrosomic infant or overweight/obese) were oversampled in this study population. Women were excluded when they were not able to read and speak Dutch. Participants who were pregnant at time of recruitment were excluded for current analysis as information on preconception dietary intake was missing. Consequently, preconception dietary intake data and blood levels of folate, vitamin D and EPA+DHA were available for 66 women. The GLIMP2 study was conducted by Wageningen University & Research between 2015 and 2017. The Medical Ethics Committee of Wageningen University & Research approved the GLIMP2 study (NL50554.081.14). All women gave their written informed consent before the start of the study.

### 2.2. Dietary Assessment

Dietary intake was assessed with a validated semi-quantitative 173- item Food-Frequency Questionnaire (FFQ) assessing habitual food and beverage intake of the previous month. The FFQ was designed and validated to estimate dietary intake of energy, macronutrients, and B-vitamins in Dutch women of reproductive age [20,21,22]. The FFQ included consumption frequencies (from once a month to several times a day) and the number of units eaten or portion sizes (e.g., slices, cups, pieces, spoons, etc.) according to Dutch household measures [23]. Food groups were created and the contribution of each food group to total dietary folate, vitamin D and EPA+DHA intake was calculated. All participants were asked to report whether they used dietary supplements. For each supplement, the frequency, number of tablets or drops, type, and brand were reported. The nutrient content of the supplements was based on the product label information as obtained from the manufacturer or Anatomical Therapeutic Chemical (ATC) code website. Total nutrient intake was obtained by summing dietary intake and supplemental intake. To account for differences in bioavailability of natural and synthetic folate, folate intake was expressed as folate equivalents (FE). Total folate intake (FE µg/day) was obtained by summing dietary folate intake (FE µg/day) + 1.7 × supplemental folic acid intake (µg/day) [24]. Based on the recommended intake of folate, vitamin D, and EPA+DHA for women in the preconception period by the Health Council of The Netherlands [6,16,18], folate intake below 680 FE μg/day, vitamin D intake below 10μg/day and EPA+DHA intake below 200 mg/day were considered as inadequate.

### 2.3. Biochemical Analyses

Fasting blood samples were obtained by venipuncture in the morning at one of the hospitals for assessing blood levels of folate, vitamin D, and EPA+DHA. Blood samples were transported in a cool storage box with a temperature around 7 °C to the laboratory of Gelderse Vallei Hospital (Ede, The Netherlands) and processed within three hours after collection. Tubes with blood plasma containing EDTA for phospholipid fatty acid composition analyses were transported to the division of Human Nutrition of Wageningen University & Research (Wageningen, The Netherlands) and stored at −80°C. Plasma folate was analysed using the Siemens Dimension Vista^®^ folate method with the Dimension Vista^®^ System, a quantitative, competitive chemiluminescence immunoassay method based on LOCI^®^ technology (Siemens Healthcare, The Hague, The Netherlands) with an inter-assay coefficient of variation (CV) of 7.0%. Plasma folate levels below 10 nmol/L were considered as folate insufficient [25]. Serum 25-hydroxyvitamin D (25(OH)D) was analysed using a validated isocratic High Performance Liquid Chromatography (HPLC) method using UV detection (Chromsystems Instruments & Chemicals HmbH, Gräfelfing, Germany) with an inter-assay CV of 4.6%. There was no cross-reactivity towards 25(OH)D2. Serum 25(OH)D levels below 50 nmol/L were considered as insufficient [26]. To assess EPA+DHA status, the fatty acid composition of EDTA plasma phospholipids were analysed in the laboratory of the division of Human Nutrition of Wageningen University & Research. After isolation of the phospholipid fraction and saponification, the fatty acids were derivatized to fatty acid methyl esters (FAMEs), which were subsequently quantified with a gas chromatograph (Hewlett Packard 5890 Series II, Hewlett Packard, Palo Alto, CA, USA) equipped with a capillary column (WCOT fused silica kolom CP WAX 58, 25 m × 0.25 mm id Chrompack CP 7717) and flame-ionization detection, using nitrogen as carrier gas. Fatty acid concentrations were reported as g/100 g FAME; the sum of all peak areas of the fatty acids identified was set to 100% [27]. For EPA+DHA, there was no generally acceptable cut-off for an optimal EPA+DHA status.

### 2.4. Covariates

Anthropometric measurements were performed by trained professionals. Body weight was determined to the nearest 0.1 kg with a calibrated balance (SECA, Hamburg, Germany), while women had to take off their shoes and empty their pockets. Body height was measured with a wall-mounted stadiometer (SECA, Hamburg, Germany) to the nearest 0.1 cm, while wearing no shoes. Body Mass Index (BMI) was calculated as body weight divided by squared body height (kg/m^2^). Data on maternal age, ethnicity (Western/non-Western), marital status (married/living together), parity (no/one or more child), educational level (low/intermediate/high), smoking habits (yes/no), and alcohol consumption (g/day) were predominantly collected using LifeLines study questionnaires [28]. Data on birth country of the participant and birth country of her biologic parents was used to determine ethnicity (Western or non-Western). Educational level was evaluated based on the highest completed education and classified into three categories: low: primary school, vocational or lower general secondary education, intermediate: higher secondary education or intermediate vocational training and high: higher vocational education or university. Intakes of vitamin B_6_ and vitamin B_12_ were obtained from the FFQ and the questionnaire on supplement use. Date of blood sampling was used to define a covariate for season (summer: May–November and winter: December–April) [15].

### 2.5. Data Analyses

Participant characteristics of the study population were reported as mean ± SD, as median (interquartile rage (IQR)), or as *n* (%) for the total population. Additionally, we made two strata: one for women who did meet recommended intake and one for women who did not meet recommended intake [6,16,18]. We did this for folate (<680 FE µg/day and ≥680 FE µg/day), vitamin D (<10 µg/day and ≥10 µg/day), and EPA+DHA recommendations (<200 mg/day and ≥200 mg/day). Independent sample *t*-tests, Chi-Square tests, and Mann Whitney U tests were used to compare characteristics between participants who met and participants who did not meet the recommendation. Spearman’s rank correlation coefficients and partial correlation coefficients were used to determine the correlation of total nutrient intake, total dietary intake, supplemental intake, and nutrient intake from the top-5 dietary sources with blood levels. Analysis of covariance (ANCOVA) was performed to compare blood levels across tertiles of nutrient intake and to calculate adjusted means with 95% confidence intervals. Additionally, *p* for trend analysis was performed using the median intake of the tertile as a continuous variable in the ANCOVA model. Restricted cubic splines were used to visualize the dose-response relationship between total nutrient intake and blood levels of folate, vitamin D, and EPA+DHA. Three knots were used at the 1st, 5th, and 9th decile of intake. Significant predictors for blood levels in linear regression models (*p* < 0.05) were included as covariates in the statistical analyses. For folate these were: season of blood sampling, total energy intake, total vitamin B6 intake and total vitamin B12 intake; for vitamin D and EPA+DHA these were: educational level, season of blood sampling, total energy intake and BMI. Statistical analyses were conducted using SAS Software Version 9.4 (SAS Institute Inc., Cary, NC, USA), except for the restricted cubic splines, which were performed using R statistical software version 3.1.1 (R Foundation for Statistical Computing, Vienna, Austria) and R Studio 1.0. (Rstudio, Boston, MA, USA). A *p*-value of ≤0.05 (two-sided) was considered statistically significant.

## 3. Results

### 3.1. Participant Characteristics

Characteristics of the total study population (*n* = 66) stratified by intake categories of folate, vitamin D, and EPA+DHA are shown in Table 1. The mean age of the total study population was 31.7 ± 4.1 years and mean BMI was 25.2 ± 4.0 kg/m^2^. Median (IQR) total folate intake was 713 (672) FE µg/day, median total vitamin D was 5.9 (8.5) µg/day, and median total EPA+DHA intake was 170 (200) mg/day. Thirty-seven women (56%) used a folic acid supplement, thirty women (46%) a vitamin D supplement, and three women (5%) a supplement containing EPA+DHA. BMI was modestly correlated with plasma folate (*r* = −0.315), but not with 25(OH)D or phospholipid EPA+DHA (*r* < 0.20). Biochemical parameters including C-reactive protein (CRP), total cholesterol, triglycerides and fasting glucose were not correlated with folate, vitamin D and EPA+DHA concentrations (*r* < 0.20).

### 3.2. Folate Intake and Status

**Figure 1:**
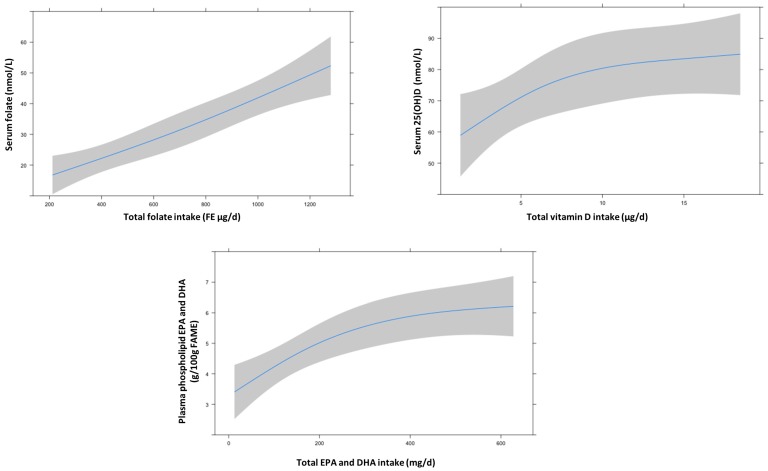
Associations between total intake and blood levels of folate, vitamin D, and EPA+DHA, obtained by restricted cubic spline regressions with three knots located at 1st, 5th, and 9th decile. Solid lines represent the estimated dose-response curves, and the shaded areas represent the 95% confidence intervals. Intake-status association of folate was adjusted for season of blood sampling, energy intake (kJ) and intake of vitamin B6 (mg/day) and B12 (μg/day) (*p* for non-linearity = 0.69); intake-status association of vitamin D was adjusted for season of blood sampling, education level (low/intermediate/high), BMI (kg/m^2^) and energy intake (kJ) (*p* for non-linearity = 0.17); intake-status association of EPA+DHA was adjusted for season of blood sampling, education level (low/intermediate/high), BMI (kg/m^2^) and energy intake (kJ) (*p* for non-linearity = 0.05).

Half of the participants met the recommended daily folate intake (≥680 FE µg; *n* = 33). Women with an adequate folate intake had a significantly higher median (IQR) total folate intake (943 (124) FE µg/d versus 272 (102) FE µg/day, *p* < 0.001), were more likely to take folate supplements (100% versus 12%, *p* < 0.001), had higher intakes of vitamin B6 (2.1 (3.4) mg/day versus 1.8 (0.8) mg/day, *p* < 0.05), higher intakes of vitamin B12 (5.4 (8.0) μg/day versus 4.3 (2.1) μg/day, *p* < 0.05), and had higher plasma folate levels (40.9 ± 18.6 nmol/L versus 17.9 ± 9.7 nmol/L, *p* < 0.001) compared to women with an inadequate folate intake (Table 1). Nine women (13.6%) had inadequate folate plasma levels. Eight of them did not meet the recommended dietary folate intake. Total median (IQR) folate intake of all participants was 713 (672) FE µg/day, of which 262 (102) FE µg/day was from dietary sources (Table 2). Vegetables (25%), bread and cereal products (22%), dairy products (10%), fruit (10%), and oils and fats (5%) were the main dietary sources of folate intake. Total folate intake (adjusted partial correlation coefficient 0.55, *p* for trend <0.001) and folate from supplements (adjusted partial correlation coefficient 0.75, *p* for trend <0.001) were significantly positively associated with plasma folate levels. None of the dietary sources of folate was significantly associated with plasma folate. The linear dose-response curve for total folate intake and plasma folate level is shown in Figure 1 (*p* for non-linearity = 0.69).

### 3.3. Vitamin D Intake and Status

Twenty-two participants (33%) had an adequate vitamin D intake (≥10 µg/day), while forty-four participants did not meet this recommendation. Women with an adequate vitamin D intake had a significantly higher median (IQR) total vitamin D intake (13.0 (2.1) µg/day versus 3.7 (3.2) µg/day, *p* < 0.001), were more likely to take vitamin D supplements (100% versus 18%, *p* < 0.001), and had higher serum 25(OH)D levels (76 ± 19 nmol/L versus 68 ± 26 nmol/L, *p* = 0.16) compared to women with an inadequate vitamin D intake (Table 1). Fifteen women (22.7%) had insufficient 25(OH)D serum levels, of which 13 had dietary intakes below the recommendation.

Total median (IQR) vitamin D intake was 5.9 (8.5) µg/day, of which 3.3 (2.0) µg/day was obtained from dietary sources (Table 3). Oils and fats (39%) were the main contributors to total dietary vitamin D intake. Fish intake was the second most important contributor (20%). Furthermore, meat (14%), eggs (10%), and dairy products (5%) belonged to the top-5 dietary sources of vitamin D intake. Total vitamin D intake (adjusted partial correlation coefficient 0.42, *p* for trend 0.04), vitamin D from supplements (adjusted partial correlation coefficient 0.40, *p* for trend 0.006), total dietary vitamin D (adjusted partial correlation coefficient 0.30, *p* for trend 0.001) and vitamin D from oils and fats (adjusted partial correlation coefficient 0.38, *p* for trend 0.02) were significantly positively associated with serum 25(OH)D levels. The dose-response between total vitamin D intake and serum 25(OH)D levels is shown in Figure 1 (*p* for linearity = 0.17).

### 3.4. EPA and DHA Intake and Status

Forty-four percent of the participants met the recommended intake of the *n*-3 fatty acids EPA+DHA (≥200 mg/day; *n* = 29); these women had a significantly higher median total EPA+DHA intake (310 (240) mg/day versus 100 (110) mg/day, *p* < 0.001) compared with those who did not meet the recommendation (Table 1). Only three women (5%) used a supplement containing EPA+DHA. Plasma phospholipid EPA+DHA levels were significantly higher in participants with an adequate EPA+DHA intake than in participants with an inadequate intake (5.9 ± 1.5 g/100 g FAME versus 4.6 ± 1.6 g/100 g FAME, *p* = 0.001). Total median (IQR) EPA+DHA intake was 170 (200) mg/day, of which 165 (190) mg/day was from dietary sources (Table 4). Fish (69%) was the main contributor to total dietary EPA+DHA intake, followed by meat (6%) and eggs (4%). Total EPA+DHA intake (adjusted partial correlation coefficient 0.67, *p* for trend 0.002), total dietary EPA+DHA (adjusted partial correlation coefficient 0.63, *p* for trend 0.001), EPA+DHA from total fish intake (adjusted partial correlation coefficient 0.67, *p* for trend < 0.001) and EPA+DHA from fatty fish (adjusted partial correlation coefficient 0.51, *p* for trend = 0.001) were significantly positively associated with plasma phospholipid EPA+DHA levels. Only three women took EPA+DHA containing supplements, therefore no p for trend could be calculated, but EPA+DHA from supplements was significantly correlated with plasma phospholipid EPA+DHA levels (adjusted partial correlation coefficient 0.38, *p* = 0.02). Figure 1 shows the nonlinear dose-response curve between total EPA+DHA intake and plasma phospholipid EPA+DHA level, with the curve flattening at higher intake levels (*p* for non-linearity = 0.05).

## 4. Discussion

In our cross-sectional study in 66 Dutch women aged 18–40 years who wished to become pregnant, 50% had an inadequate folate intake (<680 FE µg/day), 67% had an inadequate vitamin D intake (<10 µg/day), and 56% had an inadequate EPA+DHA intake (<200 mg/day) according to the recommendations given by the Health Council of the Netherlands. Dietary intakes of folate, vitamin D and EPA+DHA were significantly positively associated with their blood levels. However, 14% of the women had an inadequate folate status and 23% an inadequate vitamin D status. We observed significant associations between folate intake from supplements with plasma folate levels, whereas dietary folate intake was not associated with folate status markers. Oils and fats were the main contributors to total dietary vitamin D intake followed by fish. Significant vitamin D intake-status associations were observed for total vitamin D intake, vitamin D from supplements, dietary vitamin D and vitamin D from oils and fats with serum 25(OH)D. Fish, mainly fatty fish, was the most important contributor to dietary EPA+DHA intake. In line with this, significant associations were observed between total EPA+DHA intake, total dietary EPA+DHA intake, and EPA+DHA obtained from (fatty) fish with plasma phospholipid EPA+DHA.

### 4.1. Folate Intake

Only 50% of the women met the recommended folate intake of 680 FE µg/day. This is in line with previous research carried out among Dutch pregnant women where 31% of women with a lower socioeconomic status and 63% of the women with a higher socioeconomic status used a folic acid supplement prior to conception [29]. This is also in line with the reported folic acid supplement use of 40–51% in the preconception period in Australia [30,31]. Other studies reporting supplement use during pregnancy found that up to 95% of pregnant women used supplements, with multivitamin supplements and folic acid supplements as the most commonly reported [32,33,34]. These studies indicate that folate intake may be higher later in pregnancy. However, to reduce the risk of neural tube defects, adequate folate intake needs to be achieved in the periconceptional period [8]. Therefore, the high proportion of inadequate folate intake observed in our study remains of high concern. The strong association between total folate intake and plasma folate levels as observed in our study population is supported by a meta-analysis, using data from mostly non-pregnant and non-lactating women of childbearing age, reporting a 47% increase in plasma folate levels when doubling total folate intake [35].

Of the total folate intake reported (713 (672) FE µg/day), only 262 (102) µg/day was derived from dietary sources, predominantly vegetables and bread and cereals. The observed dietary folate intake is comparable to dietary folate intake reported in the Dutch National Food Consumption Survey (DNFCS) 2007–2010 (216 (92) µg/day for women aged 19–30 years and 242 (102) µg/day for women aged 31–50 years) [36]. A study in healthy Norwegian women aged 47–49 years and 71–74 years also found that vegetables, bread and cereals were main contributors to dietary folate intake [37]. However, in this Norwegian study, vegetables, fruit, and orange juice intake were significantly associated with plasma folate levels, whereas we did not observe this association. This may be explained by the substantial contribution of folate from supplements and relative low contribution of dietary folate to total folate intake in our population.

### 4.2. Vitamin D Intake

There has been an increasing attention on the high prevalence of inadequate vitamin D intake and vitamin D deficiency [38]. As vitamin D deficiency in the mother has been linked to various health issues in pregnancy and the offspring ref [9,10,11,12,13,14], adequate vitamin D intake in the preconception period is an important matter. However, in our study, two-thirds of the women did not meet the recommendation for vitamin D intake of ≥10 µg/dayay, while 13 women (30%) had serum 25(OH)D levels below 50 nmol/L. In the group with adequate vitamin D intake, only two out of 22 women (9%) had serum 25(OH)D levels below 50 nmol/L. The association of vitamin D intake with 25(OH)D levels was more pronounced in the winter season, and this underlines the importance of meeting the vitamin D recommendation, especially in the winter.

Of the total vitamin D intake reported in our study, 3.3 (2.0) µg/day was from dietary sources. Oils and fats were the most important dietary sources contributing to dietary vitamin D intake, followed by fish, meat, eggs, and dairy products, which is in line with a recent study in Dutch older adults [39]. The observed associations between vitamin D intake, total dietary vitamin D intake, vitamin D from supplements, and vitamin D intake from fats and oils with serum 25(OH)D levels, are in agreement with the recent study in Dutch older adults. Thus, even though sunlight is the most important source of vitamin D [15], we and Vaes (2016) showed that both dietary and supplemental vitamin D have a substantial impact on serum 25(OH)D levels. These results imply that women with inadequate vitamin D intake may obtain an adequate intake by an increased consumption of fats and oils or use of a vitamin D supplement. This is supported by a recent meta-analysis which observed that the intake of ±300 g fish per week over a period of at least four weeks is associated with an average increase of 4.4 nmol/L in 25(OH)D levels [40]. Although fish intake was the second contributor in our population, we did not observe a significant association between fish intake and 25(OH)D levels. This could be due to the fact that the median intake of ±100 g fish per week in our study was substantially lower than the average intake level of 300 g fish per week in the meta-analysis [40].

With respect to vitamin D supplement use, 45% of the women in our study used a vitamin D supplement in the preconception period, which is high compared to a study in Australia, where only 14% of the participants, also mainly highly educated, used a vitamin D supplement during the three months prior to conception [30]. This difference may have arisen due to geographical differences, with more sun exposure in Australia than the Netherlands. Furthermore, in our cohort one of the most used pregnancy multivitamin supplements contained both folate and vitamin D, which may explain the higher vitamin D supplement use in our population. In addition, it may explain why all the women with an adequate vitamin D intake also had an adequate folate intake.

### 4.3. EPA and DHA Intake

Less than half of the women in our study met the recommended 200 mg daily intake of EPA+DHA. However, the median (IQR) intake of 170 (200) mg/day in our study was higher than the intake of EPA+DHA in the general population with a median intake of 75 (92) mg/day for women aged 19–30 years and 89 (106) mg/day for women aged 31–50 years [36]. Fish, and especially fatty fish, was the main contributor to dietary EPA+DHA intake in our study population. We observed strong significant correlations of total EPA+DHA intake, total dietary EPA+DHA intake, and EPA+DHA obtained from (fatty) fish with plasma phospholipid EPA+DHA. This could imply that women with modest inadequate EPA+DHA intake may obtain an adequate intake by an increased consumption of fatty fish. This is in line with a study among adult Swedish women showing comparable strong correlations between dietary intake of EPA+DHA and EPA+DHA plasma phospholipids [41]. Only three women in our study (5%) used a supplement containing EPA+DHA, which is similar to the data of the DNFCS 2007–2010 [36] and to a cohort in Australia where 9% of the women used a fish oil supplement during the three months prior to conception [30]. The women in our study who took EPA+DHA containing supplements had higher plasma phospholipid EPA+DHA plasma levels, adjusted for dietary EPA+DHA intake, than the women who did not take a supplement, indicating that for women who cannot or do not want to consume fish, EPA+DHA containing supplements might be helpful to achieve recommended intakes and increase EPA+DHA levels.

### 4.4. Supplement Use

As demonstrated in our study, supplementation can help in achieving adequate intake levels and consequently achieve adequate blood levels of folate and vitamin D. However, some considerations should be taken into account regarding supplement use. Women who take prenatal supplements are often women with a higher educational level, who, in general, already achieve higher intakes of micronutrients [42,43]. Health policies encouraging supplement use might not reach the women with the lowest intake levels, but increase supplement consumption in health-conscious women with already adequate intake levels, who are consequently at risk of overconsumption [32]. This might especially occur in countries with mandatory fortification of bread, for example, with folate, such as in the United States, United Kingdom and Australia. It should also be noted that the WHO advises against using supplements, other than folic acid, vitamin D and iron in the preconception period or during pregnancy when not deficient, including multivitamins as there has been no proven additional benefit of other supplements and may lead to overconsumption of specific micronutrients [44].

### 4.5. Study Limitations and Strengths

Before heading to the conclusion, some limitations and strengths of this study need to be discussed. First of all, the sample size of this study was relatively small (*n* = 66). A relatively small sample size may lead to reduced power and reduced ability to detect significant associations with a small effect size. However, median (IQR) vitamin D intake from dietary sources was in line with the values described in the study of Vaes (*n* = 595) [38], indicating that our sample size was large enough to provide robust vitamin D intake estimates. Secondly, our FFQ was not validated for vitamin D and the *n*-3 fatty acids EPA and DHA, but was a FFQ which was validated for dietary intake of energy, macronutrients, and B-vitamins in Dutch women of reproductive age [20,21,22]. However, the FFQ was designed to capture energy intake (i.e., an extensive FFQ including a large variety of food items) and included food items covering the most important dietary sources of vitamin D and EPA+DHA (e.g., fat and oils, fish, meat, and dairy). Nevertheless, measurement errors resulting from aggregation of food-items in an FFQ, under- or over reporting, recall bias, seasonal influence and thus biased estimates associated with self-report methods cannot be excluded [21]. Thirdly, our study population consisted of mostly highly educated women with a Western ethnicity, with an oversampling of women with a high risk of GDM and does not represent the general population of women who wish to get pregnant. Our results may therefore be limited in generalizability to other study populations with respect to reported dietary intake estimates and prevalence of inadequacy. However, intake-status associations are less likely to be influenced by education and were adjusted for both educational level and BMI (most important GDM risk factor). Fourth, the vitamin D assay used in this study only measured 25(OH)D3 and not 25(OH)D2. Therefore, our vitamin D status estimates may be slightly lower than the actual vitamin D status. Earlier data, however, indicate that 25(OH)D2 status is only a small fraction of the total 25(OH)D status, specifically 25(OH)D2 contributed median (interquartile range) 1.4 (0.5–2.7) ng/ml to the total 25(OH)D status of 24.3 (19.2–30.1) ng/ml [45]. Therefore, we do not expect that this lack of 25(OH)D2 data substantially influenced the associations in our study. Major strengths of this study include availability of blood samples of women in the preconception period and information on women’s total nutrient intake, including both diet and supplement use, enabling us to examine intake-status associations. Because the FFQ covered dietary intake of the previous month, appropriate blood biomarkers were used, as plasma folate, serum 25(OH)D, and plasma phospholipid EPA+DHA levels were considered indicators of recent dietary intake [35,46,47]. Furthermore, potential relevant covariates appropriate to specific nutrient intakes were used in statistical analyses to reduce the risk of confounding.

## 5. Conclusions

Results of our study showed that even among highly educated women who wanted to become pregnant, a large proportion did not meet recommendations regarding folate, vitamin D and EPA+DHA intake. Significant associations were found between total folate, total vitamin D and total EPA+DHA intake and their blood levels. Women with inadequate vitamin D and EPA+DHA intake may obtain an adequate intake by an increased consumption of fats and oils and fish. In this population, supplement use contributed substantially to total folate and vitamin D intake and status levels. Promotion of fish intake and folic acid and vitamin D supplement use of for women wish to become pregnant is necessary, since intake of the top-5 dietary sources, covering at least 80% of dietary intakes of these micronutrients, cannot suffice the nutritional requirements in most women to obtain an adequate nutritional status in the preconception period. Results of our study contribute to the current scientific evidence as data on intake of folate, vitamin D, and EPA+DHA from different sources and data on nutrient status is very limited, especially among women who wish to become pregnant.

## Figures and Tables

**Table 1:** Characteristics of the total study population (*n* = 66) and stratified by meeting the recommended intake of folate, vitamin D, and EPA+DHA for women in the preconception period according to the Health Council of the Netherlands.

Characteristics	Total Population (*n* = 66)	Folate Intake < 680 FE µg/day (*n* = 33)	Folate Intake ≥ 680 FE µg/day (*n* = 33)	Vitamin D Intake < 10 µg/day (*n* = 44)	Vitamin D Intake ≥ 10 µg/day (*n* = 22)	EPA+DHA Intake < 200 mg/day (*n* = 37)	EPA+DHA Intake ≥ 200 mg/day (*n* = 29)
Maternal age (years)	31.7 ± 4.1	31.2 ± 4.7	32.1 ± 3.4	31.0 ± 4.0	33.0 ± 4.0	31.7 ± 4.5	31.7 ± 3.5
BMI (kg/m^2^)	25.2 ± 4.0	26.1 ± 4.4	24.3 ± 4.2	25.3 ± 4.5	25.0 ± 4.4	24.9 ± 4.6	25.6 ± 4.2
Western ethnicity (%)	63 (95.5%)	30 (90.9%)	33 (100%)	42 (95.5%)	21 (95.5%)	34 (91.9%)	29 (100.0%)
Marital status married (%)	55 (83.3%)	27 (81.8%)	28 (84.9%)	37 (84.1%)	18 (81.8%)	32 (86.5%)	23 (79.3%)
Parity, ≥1 child (%)	60 (90.9%)	29 (87.9%)	31 (93.9%)	39 (88.6%)	21 (95.5%)	34 (91.9%)	26 (89.7%)
Educational level (%) ^1^							
Low	3 (4.6%)	2 (6.1%)	1 (2.6%)	3 (6.8%)	0 (0.0%)	2 (5.4%)	1 (3.5%)
Intermediate	22 (33.3%)	12 (36.4%)	10 (30.3%)	14 (31.8%)	8 (36.4%)	12 (32.4%)	10 (34.5%)
High	41 (62.1%)	19 (57.6%)	22 (66.7%)	27 (61.4%)	14 (63.6%)	23 (62.2%)	18 (62.7%)
Smokers (%)	8 (12.1%)	5 (15.2%)	3 (9.1%)	6 (13.6%)	2 (9.1%)	4 (10.8%)	4 (13.8%)
Alcohol (g/day)	0.9 (4.1)	0.5 (4.1)	0.9 (3.6)	0.8 (3.8)	1.0 (4.3)	0.5 (1.5)	1.8 (3.9) *
Blood sampling between December and April (%)	10 (15.2%)	5 (15.2%)	5 (15.2%)	7 (15.9%)	3 (13.6%)	4 (10.8%)	6 (20.7%)
Energy (kJ)	8424 (2701)	8152 (2888)	8697 (2058)	8338 (2752)	8495 (2218)	8152 (2376)	8913 (2172)
Total carbohydrate (E%) ^2^	45.4 (5.8)	44.9 (6.6)	46.0 (6.3)	45.4 (7.5)	45.2 (4.6)	45.7 (6.4)	44.4 (7.7)
Total protein (E%) ^2^	15.7 (2.5)	15.9 (1.6)	14.9 (3.2)	15.4 (2.6)	15.9 (2.4)	15.8 (2.9)	15.6 ± 2.1
Total fat (E%) ^2^	36.2 (5.9)	36.2 (5.0)	36.3 (5.4)	36.2 (6.6)	36.4 (5.0)	35.1 (5.3)	37.1 (6.3)
Total folate (FE µg)	713 (672)	272 (102)	943 (124) **	323 (657)	898 (145) **	338 (700)	892 (551)
Folate supplement (%)	37 (56.1%)	4 (12.1%)	33 (100%) **	18 (40.9%)	19 (86.4%) **	16 (43.2%)	21 (72.4%) *
Total vitamin D (µg)	5.9 (8.5)	3.7 (3.0)	10.3 (7.4) **	3.7 (3.2)	13.0 (2.1) **	4.8 (8.9)	6.4 (7.0)
Vitamin D supplement (%)	30 (45.5%)	7 (21.2%)	23 (69.7%) **	8 (18.2%)	22 (100.0%) **	15 (40.5%)	15 (51.7%)
Total EPA+DHA (mg)	170 (200)	130 (150)	220 (250)	180 (160)	160 (270)	100 (110)	310 (240) **
EPA+DHA supplement (%)	3 (4.6%)	1 (3.0%)	2 (6.1%)	3 (6.8%)	0 (0.0%)	0 (0.0%)	3 (10.3%) *
Total vitamin B6 (mg)	1.9 (1.4)	1.8 (0.8)	2.1 (3.4) *	1.8 (0.7)	2.9 (4.5) **	1.8 (1.6)	1.9 (1.2)
Total vitamin B12 (µg)	4.6 (3.3)	4.3 (2.1)	5.4 (8.0) *	4.4 (2.3)	5.6 (9.8)	4.1 (2.2)	5.8 (4.7) **
Plasma folate (nmol/L)	29.4 ± 18.7	17.9 ± 9.7	40.9 ± 18.6 **	26.9 ± 18.4	34.4 ± 18.7	26.9 ± 15.5	32.6 ± 22.0
Insufficient plasma folate (<10 nmol/L)	9 (13.6%)	8 (24.2%)	1 (3.0%) *	8 (18.2%)	1 (4.5%)	4 (10.8%)	5 (17.2%)
Serum 25(OH)D (nmol/L)	70.6 ± 23.8	65.7 ± 26.2	75.5 ± 20.5	67.6 ± 25.7	76.5 ± 18.5	68.6 ± 22.9	73.1 ± 25.5
Insufficient serum 25(OH)D (<50 nmol/L)	15 (22.7%)	10 (30.3%)	5 (15.2%)	13 (29.6%)	2 (9.1%)	9 (24.3%)	6 (20.7%)
Plasma phospholipid EPA+DHA (g/100 g FAME)	5.2 ± 1.7	5.0 ± 1.6	5.3 ± 1.8	5.2 ± 1.8	5.0 ± 1.5	4.6 ± 1.6	5.9 ± 1.5 **

25(OH)D 25-hydroxyvitamin D; EPA eicosapentaenoic acid; DHA docosahexaenoic acid; FAME Fatty Acid Methyl Esters. Data are presented as mean ± standard deviation, as median (interquartile range), or as *n* (%). Subgroups were created based on the recommended intake of folate, vitamin D, and EPA+DHA for women in the preconception period according to the Health Council of the Netherlands. Independent *t*-tests, Chi-Square tests, and Mann Whitney U tests were performed between the group below and above the recommended nutrient intake of interest (* *p* ≤ 0.05, ** *p* ≤ 0.01). ^1^ Low educational level: primary school, vocational or lower general secondary education; intermediate educational level: higher secondary education or intermediate vocational training; high educational level: higher vocational education or university. ^2^ E%: the amount of energy derived from that nutrient.

**Table 2:** Absolute (µg/day) and relative (%) contribution of dietary sources to total dietary folate intake, Spearman’s rank and partial correlation coefficients (R) between folate intake and plasma folate levels, and adjusted means with 95% confidence intervals for plasma folate levels (nmol/L) according to tertiles of folate intake (µg/day) among Dutch women with a pregnancy wish (*n* = 66).

	Contribution		Correlation		Adjusted Means ^4^ with 95%CIs			*p* for Trend
	µg/day ^1^	%	R ^2^	R ^3^	Tertile 1	Tertile 2	Tertile 3	
Total folate intake	713 (672)		0.58 **	0.55 **	≤294	295–894	≥895	<0.001
Plasma folate					17.6	28.6	42.0	
					(10.6–24.7)	(21.7–35.4)	(34.8–49.2)	
Folate from supplements	340 (680)		0.68 **	0.67 **	≤0	0.1–679	≥680	<0.001
Plasma folate					17.6	29.7	40.7	
					(12.2–23.0)	(19.1–40.3)	(35.5–46.0)	
Total dietary folate	262 (102)	100	−0.08	−0.20	≤223	224–293	≥294	0.257
Plasma folate					31.4	30.2	26.5	
					(25.5–37.3)	(24.5–35.9)	(20.6–32.5)	
Folate from vegetables	63.2 (50.2)	25	0.06	0.04	≤47.9	48.0–83.3	≥83.4	0.324
Plasma folate					30.1	25.2	33.1	
					(24.6–35.6)	(19.8–30.7)	(27.6–38.6)	
Folate from bread and cereal products ^5^	57.5 (39.3)	22	−0.10	−0.19	≤46.0	46.1–69.5	≥69.6	0.763
Plasma folate					29.9	29.7	28.6	
					(24.3–35.5)	(24.0–35.4)	(22.8–34.4)	
Folate from dairy products ^6^	26.8 (24.0)	10	0.01	−0.15	≤18.7	18.8–34.0	≥34.1	0.355
Plasma folate					32.6	27.3	28.5	
					(26.6–38.6)	(21.8–32.9)	(22.2–34.8)	
Folate from fruit	25.6 (22.4)	10	0.13	−0.14	≤17.6	17.7–30.7	≥30.8	0.502
Plasma folate					29.5	32.0	26.5	
					(23.5–35.5)	(26.5–37.5)	(20.4–32.6)	
Folate from oils and fats ^7^	0.27 (3.32)	5	−0.23	−0.20	≤0.07	0.08–0.63	≥0.64	0.102
Plasma folate					32.0	30.7	25.4	
					(26.4–37.7)	(25.0–36.4)	(19.7–31.2)	

Folate intake (FE µg/day), Plasma folate (nmol/L), ** *p* ≤ 0.01. ^1^ Median (IQR). ^2^ Spearman’s rank correlation with plasma folate (nmol/L) as dependent variable. ^3^ Partial correlation with plasma folate (nmol/L) as dependent variable: adjusted for season of blood sampling, total energy intake (kJ), intake of total vitamin B_6_ and vitamin B12. For supplemental folate, additional adjustment was done for dietary folate intake; for dietary folate, additional adjustment was done for folate intake from supplement; for folate from top-5 dietary sources additional adjustment for folate intake from other selected dietary sources (i.e., vegetables, bread and cereal products, dairy products, fruit, and oils and fats) was done. ^4^ Means were adjusted for the same covariates as for partial correlation, and calculated with ANCOVA. ^5^ includes bread, breakfast cereals, pasta, and rice. ^6^ includes milk, yoghurt drinks, cheese, yoghurt, fromage frais, coffee creamer, and ice cream. ^7^ includes liquid, soft and hard cooking fats and margarine, and vegetable oils.

**Table 3:** Absolute (µg/day) and relative (%) contribution of dietary sources to total dietary vitamin D intake, Spearman’s rank and partial correlation coefficients (R) between vitamin D intake and serum 25(OH)D levels, and adjusted means with 95% confidence intervals for serum 25(OH)D levels (nmol/L) according to tertiles of vitamin D intake (µg/day) among Dutch women with a pregnancy wish (*n* = 66).

	Contribution		Correlation		Adjusted Means ^4^ with 95%CIs			*p* for Trend
	µg/day ^1^	%	R ^2^	R ^3^	Tertile 1	Tertile 2	Tertile 3	
Total vitamin D	5.9 (8.5)		0.32 **	0.42 **	≤3.6	3.7–10.2	≥10.3	0.04
Serum 25(OH)D					62.5	71.2	78.0	
					(52.6–72.5)	(61.5–80.9)	(68.4–87.5)	
Vitamin D from supplements	0 (7.5)		0.30 *	0.40 **	≤0	0.1–4.9	≥5.0	0.006
Serum 25(OH)D					64.5	55.6	79.4	
					(57.6–71.5)	(24.8–86.3)	(71.5–87.3)	
Total dietary vitamin D	3.3 (2.0)	100	0.12	0.30 *	≤2.8	2.9–3.8	≥3.9	0.001
Serum 25(OH)D					57.4	71.5	82.8	
					(48.2–66.6)	(62.8–80.2)	(72.9–92.6)	
Vitamin D from oils and fats ^5^	1.1 (2.0)	39	0.20	0.38 **	≤0.6	0.7–2.0	≥2.1	0.02
Serum 25(OH)D					60.6	69.6	80.7	
					(50.2–71.1)	(60.8–78.3)	(70.1–91.3)	
Vitamin D from fish	0.53 (0.76)	20	−0.04	0.18	≤0.2	0.3–0.7	≥0.8	0.18
Serum 25(OH)D					63.3	73.8	74.1	
					(53.5–73.2)	(65–82.6)	(64.8–83.3)	
Vitamin D from meat	0.43 (0.37)	14	0.09	0.03	≤0.3	0.4–0.6	≥0.7	0.43
Serum 25(OH)D					69.2	69.5	73.0	
					(59.6–78.8)	(59.9–79)	(63.1–83.0)	
Vitamin D from egg	0.23 (0.35)	10	−0.14	−0.07	≤0.1	0.2–0.3	≥0.4	0.96
Serum 25(OH)D					72.0	67.5	71.8	
					(62.1–81.9)	(57.4–77.6)	(61.7–81.9)	
Vitamin D from dairy products ^6^	0.12 (0.11)	5	−0.06	0.13	≤0.01	0.02–0.10	≥0.11	0.11
Serum 25(OH)D					74.0	63.7	73.5	
					(65.0–83.0)	(54.0–73.4)	(63.8–83.2)	

25(OH)D 25-hydroxyvitamin D. Vitamin D intake (µg/day), and serum 25(OH)D (nmol/L), * *p* ≤ 0.05, ** *p* ≤ 0.01. ^1^ Median (IQR). ^2^ Spearman’s rank correlation with serum 25(OH)D (nmol/L) as dependent variable. ^3^ Partial correlation with serum 25(OH)D as dependent variable: adjusted for season of blood sampling, education level (low/intermediate/high), BMI (kg/m^2^) and energy intake (kJ). For supplemental vitamin D additionally adjusted for dietary vitamin D intake; for dietary vitamin D additionally adjusted for vitamin D intake from supplement; for vitamin D from top-5 dietary sources additional adjustment for vitamin D intake from other selected dietary sources (i.e., oils and fats, fish, meat, egg, and dairy products). ^4^ Means were adjusted for the same covariates as for partial correlation, and calculated with ANCOVA. ^5^ includes liquid, soft and hard cooking fats and margarine, and vegetable oils. ^6^ includes milk, yoghurt drinks, cheese, yoghurt, fromage frais, coffee creamer, and ice cream.

**Table 4:** Absolute (mg/day) and relative (%) contribution of dietary sources to total EPA and DHA intake, Spearman’s rank and partial correlation coefficients between EPA and DHA intake and plasma phospholipid EPA and DHA, and adjusted means with 95% confidence intervals for plasma phospholipid EPA and DHA (g/100 g FAME) according to tertiles of EPA and DHA intake (mg/day) among Dutch women with a pregnancy wish (*n* = 66).

	Contribution		Correlation		Adjusted Means^4^ with 95%CIs			*p* for Trend
	µg/day ^1^	%	R ^2^	R ^3^	Tertile 1	Tertile 2	Tertile 3	
Total EPA+DHA intake	170 (200)		0.63 **	0.67 **	<100	100–240	>240	0.002
PPL g/100 g FAME					4.1	5.3	6.0	
					(3.4–4.8)	(4.6–6.0)	(5.4–6.7)	
EPA+DHA from supplements	0 (0)		0.24	0.38 *	0	1–500	- ^a^	- ^a^
PPL g/100 g FAME					5.0	7.9		
					(4.6–5.4)	(6.1–9.7)		
Total dietary EPA+DHA	165 (190)	100	0.59 **	0.63 **	<100	100–230	>230	0.001
PPL g/100 g FAME					4.2	5.5	5.9	
					(3.5–4.8)	(4.8–6.2)	(5.2–6.5)	
EPA+DHA from fish	135 (190)	69	0.60 **	0.67 **	<70	70–90	>190	<0.001
PPL g/100 g FAME					3.8	5.9	6.0	
					(3.2–4.4)	(5.2–6.5)	(5.4–6.5)	
EPA+DHA from fatty fish	105 (170)	46	0.60 **	0.51 **	<20	20–160	>160	0.009
PPL g/100 g FAME					4.4	5.4	5.7	
					(3.7–5)	(4.7–6)	(5–6.4)	
EPA+DHA from lean fish	25 (40)	18	0.22	0.10	<10	10–30	>30	0.408
PPL g/100 g FAME					4.9	5.3	5.3	
					(4.2–5.5)	(4.7–6.0)	(4.6–6.0)	
EPA+DHA from shell fish	0 (10)	5	0.32 *	0.24	0	1–140	- ^a^	- ^a^
PPL g/100 g FAME					4.9	6.0		
					(4.4–5.3)	(5.2–6.8)		
EPA+DHA from meat	10 (10)	6	−0.06	−0.30 *	0	1–30	- ^a^	- ^a^
PPL g/100 g FAME					5.6	4.8		
					(5–6.1)	(4.3–5.3)		

EPA eicosapentaenoic acid; DHA docosahexaenoic acid; FAME fatty acid methyl esters; PPL plasma phospholipid. EPA+DHA intake (mg/day), and plasma phospholipid EPA+DHA (g/100 g FAME), * *p* ≤ 0.05, ** *p* ≤ 0.01. ^1^ Median (IQR). ^2^ Spearman’s rank correlation with plasma phospholipid EPA+DHA (g/100 g FAME) as dependent variable. ^3^ Partial correlation with plasma phospholipid EPA+DHA (g/100 g FAME) as dependent variable: adjusted for season of blood sampling, education level (low/intermediate/high), BMI (kg/m^2^) and energy intake (kJ). For supplemental EPA+DHA additionally adjusted for dietary EPA+DHA intake; for dietary EPA+DHA additionally adjusted for EPA+DHA intake from supplement; for EPA+DHA from top-5 dietary sources additional adjustment for EPA+DHA intake from other selected dietary sources (i.e., fish (fatty, lean and shell fish), meat, and egg). ^4^ Means were adjusted for the same covariates as for partial correlation, and calculated with ANCOVA. ^a^ Due to the low amount of EPA+DHA in lean fish, shellfish, meat, and egg, two groups were made instead of tertiles and no p for trend could be calculated.

## References

[1] Abu-Saad K., Fraser D. (2010). Maternal nutrition and birth outcomes. Epidemiol. Rev..

[2] Emmett P.M., Jones L.R., Golding J. (2015). Pregnancy diet and associated outcomes in the Avon longitudinal study of parents and children. Nutr. Rev..

[3] Langley-Evans S.C. (2015). Nutrition in early life and the programming of adult disease: A review. J. Hum. Nutr. Diet..

[4] Parisi F., Laoreti A., Cetin I. (2014). Multiple micronutrient needs in pregnancy in industrialized countries. Ann. Nutr. Metab..

[5] Huijgen N.A., van de Kamp M.E., Twigt J.M., de Vries J.H., Eilers P.H., Steegers E.A., Laven J.S., Steegers-Theunissen R.P. (2014). The preconception dietary risk score; a simple tool to assess an inadequate habitual diet for clinical practice. e-SPEN J..

[6] Health Council of the Netherlands (2007). Preconception Care: A Good Beginning.

[7] Van der Meer I.M., Karamali N.S., Boeke A.J.P., Lips P., Middelkoop B.J., Verhoeven I., Wuister J.D. (2006). High prevalence of vitamin d deficiency in pregnant non-western women in the Hague, Netherlands. Am. J. Clin. Nutr..

[8] De-Regil L.M., Fernández-Gaxiola A.C., Dowswell T., Peña-Rosas J.P. (2010). Effects and safety of periconceptional folate supplementation for preventing birth defects. Cochrane Database Syst. Rev..

[9] Bodnar L.M., Catov J.M., Simhan H.N., Holick M.F., Powers R.W., Roberts J.M. (2007). Maternal vitamin D deficiency increases the risk of preeclampsia. J. Clin. Endocrinol. Metab..

[10] Zhang C., Qiu C., Hu F.B., David R.M., Van Dam R.M., Bralley A., Williams M.A. (2008). Maternal plasma 25-hydroxyvitamin d concentrations and the risk for gestational diabetes mellitus. PLoS ONE.

[11] Morley R., Carlin J., Pasco J., Wark J., Ponsonby A. (2009). Maternal 25-hydroxyvitamin D concentration and offspring birth size: Effect modification by infant vdr genotype. Eur. J. Clin. Nutr..

[12] Javaid M., Crozier S., Harvey N., Gale C., Dennison E., Boucher B., Arden N., Godfrey K., Cooper C. (2006). Maternal vitamin D status during pregnancy and childhood bone mass at age 9 years: A longitudinal study. Lancet.

[13] Camargo C.A., Rifas-Shiman S.L., Litonjua A.A., Rich-Edwards J.W., Weiss S.T., Gold D.R., Kleinman K., Gillman M.W. (2007). Maternal intake of vitamin D during pregnancy and risk of recurrent wheeze in children at 3 y of age. Am. J. Clin. Nutr..

[14] Devereux G., Litonjua A.A., Turner S.W., Craig L.C., McNeill G., Martindale S., Helms P.J., Seaton A., Weiss S.T. (2007). Maternal vitamin D intake during pregnancy and early childhood wheezing. Am. J. Clin. Nutr..

[15] Brouwer-Brolsma E.M., Vaes A.M., van der Zwaluw N.L., van Wijngaarden J.P., Swart K.M., Ham A.C., van Dijk S.C., Enneman A.W., Sohl E., van Schoor N.M. (2016). Relative importance of summer sun exposure, vitamin D intake, and genes to vitamin D status in dutch older adults: The b-proof study. J. Steroid Biochem. Mol. Biol..

[16] Health Council of the Netherlands (2012). Evaluation of the Dietary Reference Values for Vitamin D.

[17] Uauy R., Dangour A.D. (2006). Nutrition in brain development and aging: Role of essential fatty acids. Nutr. Rev..

[18] Health Council of the Netherlands (2015). Richtlijnen Goede Voeding 2015.

[19] Netherlands Nutrition Centre (2016). Richtlijnen Schijf van vijf Netherlands.

[20] Verkleij-Hagoort A.C., de Vries J.H., Stegers M.P., Lindemans J., Ursem N.T., Steegers-Theunissen R.P. (2007). Validation of the assessment of folate and vitamin B_12_ intake in women of reproductive age: The method of triads. Eur. J. Clin. Nutr..

[21] Siebelink E., Geelen A., de Vries J.H. (2011). Self-reported energy intake by FFQ compared with actual energy intake to maintain body weight in 516 adults. Br. J. Nutr..

[22] Streppel M.T., de Vries J.H., Meijboom S., Beekman M., de Craen A.J., Slagboom P.E., Feskens E.J. (2013). Relative validity of the food frequency questionnaire used to assess dietary intake in the Leiden Longevity Study. Nutr. J..

[23] Stichting Nederlands Voedingsstoffenbestand (2006). NEVO-Tabel: Nederlands Voedingsstoffenbestand 2006.

[24] Institute of Medicine (1998). Dietary Reference Intakes for Thiamin, Riboflavin, Niacin, Vitamin B6, Folate, Vitamin B12, Pantothenic Acid, Biotin, and Choline.

[25] Health Council of the Netherlands (2008). Towards an Optimal Use of Folic Acid.

[26] Health Council of the Netherlands (2008). Towards an Adequate Intake of Vitamin D.

[27] Glatz J., Soffers A., Katan M.B. (1989). Fatty acid composition of serum cholesteryl esters and erythrocyte membranes as indicators of linoleic acid intake in man. Am. J. Clin. Nutr..

[28] Scholtens S., Smidt N., Swertz M.A., Bakker S.J., Dotinga A., Vonk J.M., van Dijk F., van Zon S.K., Wijmenga C., Wolffenbuttel B.H. (2015). Cohort profile: Lifelines, a three-generation cohort study and biobank. Int. J. Epidemiol..

[29] De Walle H.E., de Jong-van den Berg L.T. (2008). Ten years after the dutch public health campaign on folic acid: The continuing challenge. Eur. J. Clin. Pharmacol..

[30] Livock M., Anderson P.J., Lewis S., Bowden S., Muggli E., Halliday J. (2017). Maternal micronutrient consumption periconceptionally and during pregnancy: A prospective cohort study. Public Health Nutr..

[31] McKenna E., Hure A., Perkins A., Gresham E. (2017). Dietary supplement use during preconception: The Australian longitudinal study on women’s health. Nutrients.

[32] Dubois L., Diasparra M., Bedard B., Colapinto C.K., Fontaine-Bisson B., Morisset A.S., Tremblay R.E., Fraser W.D. (2017). Adequacy of nutritional intake from food and supplements in a cohort of pregnant women in Quebec, Canada: The 3D cohort study (Design, Develop, Discover). Am. J. Clin. Nutr..

[33] Arkkola T., Uusitalo U., Pietikainen M., Metsala J., Kronberg-Kippila C., Erkkola M., Veijola R., Knip M., Virtanen S.M., Ovaskainen M.L. (2006). Dietary intake and use of dietary supplements in relation to demographic variables among pregnant Finnish women. Br. J. Nutr..

[34] Gomez M.F., Field C.J., Olstad D.L., Loehr S., Ramage S., McCargar L.J., Team A.P.S. (2015). Use of micronutrient supplements among pregnant women in Alberta: Results from the Alberta Pregnancy Outcomes and nutrition (APrON) cohort. Matern. Child Nutr..

[35] Berti C., Fekete K., Dullemeijer C., Trovato M., Souverein O.W., Cavelaars A., Dhonukshe-Rutten R., Massari M., Decsi T., Van’T Veer P. (2012). Folate intake and markers of folate status in women of reproductive age, pregnant and lactating women: A meta-analysis. J. Nutr. Metab..

[36] Van Rossum C.T.M., Fransen H.P., Verkaik-Kloosterman J., Buurma-Rethans E.J.M., Ocké M. (2011). Dutch National Food Consumption Survey 2007–2010: Diet of Children and Adults Aged 7 to 69 Years.

[37] Brevik A., Vollset S.E., Tell G.S., Refsum H., Ueland P.M., Loeken E.B., Drevon C.A., Andersen L.F. (2005). Plasma concentration of folate as a biomarker for the intake of fruit and vegetables: The hordaland homocysteine study. Am. J. Clin. Nutr..

[38] Blumfield M.L., Hure A.J., Macdonald-Wicks L., Smith R., Collins C.E. (2013). A systematic review and meta-analysis of micronutrient intakes during pregnancy in developed countries. Nutr. Rev..

[39] Vaes A., Brouwer-Brolsma E., van der Zwaluw N., van Wijngaarden J., Berendsen A., van Schoor N., van der Velde N., Uitterlinden A., Lips P., Dhonukshe-Rutten R. (2016). Food sources of vitamin D and their association with 25-hydroxyvitamin D status in dutch older adults. J. Steroid Biochem. Mol. Biol..

[40] Lehmann U., Gjessing H.R., Hirche F., Mueller-Belecke A., Gudbrandsen O.A., Ueland P.M., Mellgren G., Lauritzen L., Lindqvist H., Hansen A.L. (2015). Efficacy of fish intake on vitamin D status: A meta-analysis of randomized controlled trials. Am. J. Clin. Nutr..

[41] Lemming E.W., Nälsén C., Becker W., Ridefelt P., Mattisson I., Lindroos A.K. (2015). Relative validation of the dietary intake of fatty acids among adults in the Swedish national dietary survey using plasma phospholipid fatty acid composition. J. Nutr. Sci..

[42] Aronsson C.A., Vehik K., Yang J., Uusitalo U., Hay K., Joslowski G., Riikonen A., Ballard L., Virtanen S.M., Norris J.M. (2013). Use of dietary supplements in pregnant women in relation to sociodemographic factors—A report from the environmental determinants of diabetes in the young (teddy) study. Public Health Nutr..

[43] Pouchieu C., Levy R., Faure C., Andreeva V.A., Galan P., Hercberg S., Touvier M. (2013). Socioeconomic, lifestyle and dietary factors associated with dietary supplement use during pregnancy. PLoS ONE.

[44] World Health Organization (2016). Who Recommendations on Antenatal Care for a Positive Pregnancy Experience.

[45] Tolppanen A., Fraser A., Fraser W., Lawlor D. (2012). Risk factors for variation in 25-Hydroxyvitamin D_3_ and D_2_ concentrations and vitamin D deficiency in children. J. Clin. Endocrinol. Metab..

[46] Saadatian-Elahi M., Slimani N., Chajès V., Jenab M., Goudable J., Biessy C., Ferrari P., Byrnes G., Autier P., Peeters P.H. (2009). Plasma phospholipid fatty acid profiles and their association with food intakes: Results from a cross-sectional study within the European prospective investigation into cancer and nutrition. Am. J. Clin. Nutr..

[47] Fekete K., Marosvölgyi T., Jakobik V., Decsi T. (2009). Methods of assessment of n–3 long-chain polyunsaturated fatty acid status in humans: A systematic review. Am. J. Clin. Nutr..

